# Semi-Supervised Text Classification Framework: An Overview of Dengue Landscape Factors and Satellite Earth Observation

**DOI:** 10.3390/ijerph17124509

**Published:** 2020-06-23

**Authors:** Zhichao Li, Helen Gurgel, Nadine Dessay, Luojia Hu, Lei Xu, Peng Gong

**Affiliations:** 1 Ministry of Education Key Laboratory for Earth System Modeling, Department of Earth System, Science, Tsinghua University, Beijing 100084, China; zhichaoli@mail.tsinghua.edu.cn (Z.L.); xulei@icdc.cn (L.X.); 2 Department of Geography, University of Brasilia (UnB), Brasilia CEP 70910-900, Brazil; helengurgel@unb.br; 3 International Joint Laboratory Sentinela, FIOCRUZ, UnB, IRD, Rio de Janeiro RJ-21040-900, Brazil; nadine.dessay@ird.fr; 4 IRD, UM, UR, UG, UA, UMR ESPACE-DEV, 34090 Montpellier, France; 5 Qian Xuesen Laboratory of Space Technology, China Academy of Space Technology, Beijing 100094, China; huluojia@qxslab.cn; 6 Center for Healthy Cities, Institute for China Sustainable Urbanization, Tsinghua University, Beijing 100084, China

**Keywords:** dengue, landscape, satellite Earth observation, deep active learning, natural language processing

## Abstract

In recent years there has been an increasing use of satellite Earth observation (EO) data in dengue research, in particular the identification of landscape factors affecting dengue transmission. Summarizing landscape factors and satellite EO data sources, and making the information public are helpful for guiding future research and improving health decision-making. In this case, a review of the literature would appear to be an appropriate tool. However, this is not an easy-to-use tool. The review process mainly includes defining the topic, searching, screening at both title/abstract and full-text levels and data extraction that needs consistent knowledge from experts and is time-consuming and labor intensive. In this context, this study integrates the review process, text scoring, active learning (AL) mechanism, and bidirectional long short-term memory (BiLSTM) networks, and proposes a semi-supervised text classification framework that enables the efficient and accurate selection of the relevant articles. Specifically, text scoring and BiLSTM-based active learning were used to replace the title/abstract screening and full-text screening, respectively, which greatly reduces the human workload. In this study, 101 relevant articles were selected from 4 bibliographic databases, and a catalogue of essential dengue landscape factors was identified and divided into four categories: land use (LU), land cover (LC), topography and continuous land surface features. Moreover, various satellite EO sensors and products used for identifying landscape factors were tabulated. Finally, possible future directions of applying satellite EO data in dengue research in terms of landscape patterns, satellite sensors and deep learning were proposed. The proposed semi-supervised text classification framework was successfully applied in research evidence synthesis that could be easily applied to other topics, particularly in an interdisciplinary context.

## 1. Introduction

According to the World Health Organism (WHO), dengue affects over half of the global population, with an estimated 100–400 million infections each year worldwide [1]. In recent years, dengue has been transmitted to new geographical areas in the world, and dengue epidemics are increasing in frequency and magnitude [2].

The spatial concentration and diffusion of dengue vectors/cases can be affected by weather conditions and landscape factors (e.g., vegetation, transport, urbanization) at different spatial scales (e.g., global, regional, and local scales) [3,4,5]. The advances in satellite Earth observation (EO) readily benefit the identification of dengue landscape factors by providing a better monitoring of the Earth’s surface at different spatio-temporal scales, and entomological/epidemiological dengue risk mapping benefits from the use of satellite EO data [5]. In practice, satellite EO data, combined with weather data, can be used to predict the likelihood of future dengue epidemics so that preventative measures can be taken in advance, such as eliminating mosquito-breeding sites. Compared with weather factors, landscape factors are often more complex as landscape is often related to the vectorial capacity through vector resting and breeding sites, human–vector encounters or human mobility in different geographic contexts and at different spatial scales [6]. Several important reviews have covered such information, for example, Parselia et al. [7] proposed a scoping review that identified studies using satellite EO data for epidemiological modeling of malaria, dengue and West Nile Virus (WNV) published from 2012 to 2018. However, only 15 studies were identified for dengue where satellite EO data were used to identify meteorological and environmental factors. Sallam et al. [8] proposed a systematic review that summarized land cover, meteorological and socioeconomic factors of *Aedes* habitats, referring to dengue vectors. Moreover, our previous mapping review [9] focused on the dengue transmission in urban landscapes, and urban landscape factors derived from satellite EO data, Geographic Information System (GIS) techniques and survey questionnaires; spatial scales and dengue–landscape relationships were identified from 78 relevant studies published from inception to 31 December 2019. Despite all this, there is still a lack of overview on satellite EO data and landscape factors that could be of benefit to science and society by guiding future studies of disease risk prediction and improving health decision-making at different spatial scales (e.g., from global to local).

Information updates can be simply conducted by re-running the process of review, which would mainly include defining the research question, searching for and removing duplicates, title abstract screening, full-text eligibility and inclusion [10,11]. However, the selection of relevant studies is time-consuming and is highly dependent on the perception of reviewers, especially for title abstract screening and full-text eligibility. Under such constrained circumstances, text classification appears particularly relevant. As a typical topic in natural language processing (NLP), multiple algorithms in text classification have proved to be efficient in replacing the manual evaluation of bibliographic records (e.g., titles and/or abstracts) and reducing human workload, such as term weighting [12] and multiple machine learning (ML) algorithms [13,14,15]. Recent advances in deep learning (DL) based on convolutional neutral networks (CNNs) and recurrent neural networks (RNNs) have been used in text classification [16,17,18]. Since text classification can be considered as one sequential modelling task, RNNs have been used more frequently because of their specificity for sequential modelling tasks [16]. One kind of RNN, the long short-term memory (LSTM) performs well in text classification because it can effectively solve the problems of exploding and vanishing gradients and capture long-term dependencies in text [19]. The bidirectional LSTM (BiLSTM) is a development of the LSTM and combines forward hidden and backward hidden layers that often work better than LSTM in text classification [16]. However, when applying the algorithms above, we need to label sufficiently good-quality samples for training and validating models, which is quite time-consuming. However, deep active learning (DAL), integrating active learning (AL) in DL architecture, is able to achieve text classification based on few labelled data which can minimize the work of human labelling [20,21,22]. It would seem to be more appropriate to implement text classification based on a new bibliographic dataset for selecting relevant records, while the labelled data derived from active learning could be used as training data to train the DL architecture [22].

In this context, focusing on landscape factors affecting dengue transmission and satellite EO data currently used for identifying landscape factors, this study proposes to build a semi-supervised classification framework of literature by integrating the review process and text classification algorithms and provides an overview of dengue landscape factors and satellite EO data. The proposed framework allows for rational and effective selection of literature relevant to our objective from bibliographic databases.

## 2. Towards a Semi-Supervised Classification Framework of Literature

**Figure 1:**
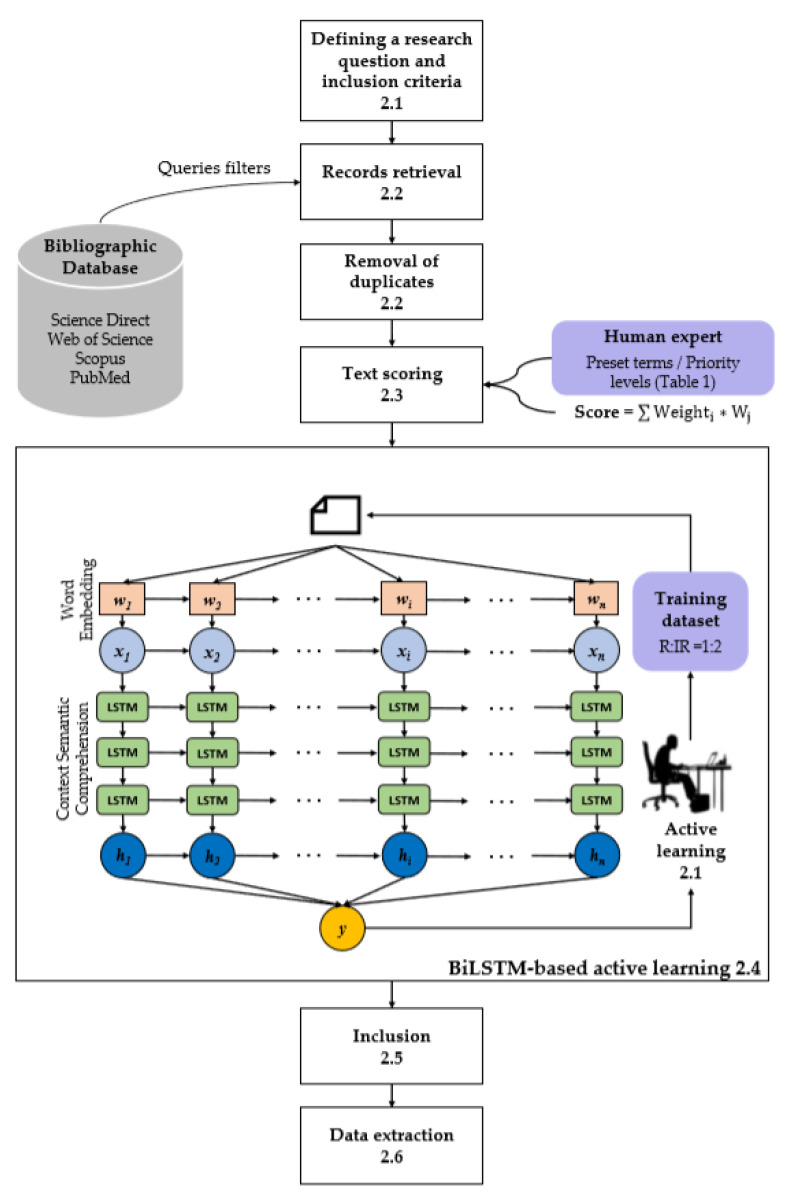
The overall workflow of semi-supervised text classification.

The framework of semi-supervised text classification integrating the review process and semi-automatic text classification (Figure 1), includes: (1) defining the research question and specifying the inclusion criteria (Section 2.1); (2) conducting a board search and removing the duplicates (Section 2.2); (3) screening titles and abstracts based on text scoring (Section 2.3); (4) preparing relevant and irrelevant samples, and conducting the BiLSTM-based active learning (Section 2.4); (5) verifying the performance of text scoring and BiLSTM-based active learning (Section 2.5); and (6) extracting dengue landscape factors and satellite EO data and charting the results (Section 2.6).

To implement the text scoring in step 3, it is necessary to remove the records that are definitively irrelevant to our topic, which also reduces the amount of data for the BiLSTM-based active learning in step 4. It should be noted that the BiLSTM model was developed and implemented based on titles and abstracts that are different from the full-text assessment in the eligibility step of the review. The detailed information is presented hereafter and no ethics approval is needed as this method is based on published journal articles.

### 2.1. Research Question and Inclusion Criteria

The objective of this study is to provide an overview on landscape factors related to dengue transmission and satellite EO data used in the identification of dengue landscape factors. Relevant records should satisfy the following criteria: (1) being an original journal article published in English; (2) highlighting landscape factors derived from satellite EO data or geographic information system (GIS) techniques; (3) being applied to dengue cases or dengue vectors; (4) modelling or correlating dengue with landscape factors. These were defined based on our objective and expert knowledge, and were used for text scoring and record sample selection for BiLSTM models.

### 2.2. Board Searches and Removal of Duplicates

The searches were performed from inception to 31 December 2019 in four databases: Science Direct, Web of Science, PubMed and Scopus, by considering the titles and abstracts of English journal articles. The queries were formed by combining dengue-related terms (i.e., dengue and *Aedes*) and the words related to “remote sensing”, “landscape” and “weather” (i.e., remote sensing, satellite, earth observation, landscape, land cover, land use, household, dwelling, habitation, precipitation and temperature) using the Boolean operator “AND” (see more details in Table A1). All search records were combined together and the duplicate records were removed using the MySQL database. The remaining records were organized in alphabetical order for further analysis.

### 2.3. Text Scoring

To efficiently eliminate the definitely-irrelevant records, we used text weighting and text scoring for ranking all the records. First, we pre-set some terms KEY*_i_* (*i* = 1, …, m) and their priority levels (i.e., high, medium and low) (Table 1) according to the criteria in Section 2.1. Each of them was randomly assigned a weight value WEIGHT*_i_* (*i* = 1, …, m) from the interval of weights that was set according to its priority level. The higher the priority level of a term, the greater its weight value. We then extracted the key terms K*_j_* (*j* = 1, …, n) and their corresponding weight values W*_j_* (*j* = 1, 2, …, n) from the title and abstract using the Natural Language Toolkit (NLTK) in Python. If K*_j_* contains pre-set terms in KEY*_i_*, we calculated the score of a record as Score = $\sum^{\;}{\mathrm{WEIGHT}}_{i}{*W}_{j}$ (i = 1, …, m; j = 1, …, n). For example, through keyword extraction using NLTK, a bibliographic record has two key terms “dengue” and “satellite”, and their weights are W(dengue) and W(satellite). According to Table 1, the weights of these two terms were randomly assigned to 8 and 5. In this case, the score of this text is W (dengue)*8 + W (satellite)*5.

All the records were then ranked in decreasing order according to the scores, and the top 1000 records were selected and merged into a subset denoted as U*_k_*. Finally, we iterated the second step 20 times, and the records in the 20 subsets U*_k_* (*k* = 1, …, 20) were combined together, and were used for the next analysis. It should be noted that random assignment of weights allows multiple iterations of text scoring that should make the results more reliable.

### 2.4. BiLSTM-Based Active Learning

To efficiently and accurately select relevant records in the absence of sufficient labelled samples, we performed a BiLSTM-based active learning based on the titles and abstracts of the records derived from text scoring (Figure 1).

Prior to training the BiLSTM model (see more details in Appendix C) [23], we created an initial training dataset by selecting 15 relevant samples and 30 irrelevant samples from the results of text scoring based on the criteria in Section 2.1. The initial training dataset was used to train the BiLSTM model.

Based on the word embedding derived from the unlabelled data using the Word2Vec CBOW model [24] (see more details in Appendix B), the BiLSTM model was used to identify the “potential” records from unlabelled data, which were then manually labelled as either relevant or irrelevant based on the four criteria in Section 2.1. Meanwhile, we improved the training dataset by combining the selected relevant records and previous relevant samples, and randomly selected irrelevant records from the results of text scoring in order to keep the ratio of relevant and irrelevant samples at 1:2. Finally, the BiLSTM model was re-trained using the new training dataset to identify the potential citations from the remaining unlabelled data. The parameters of the BiLSTM architecture were updated by training the results from the previous round. BiLSTM learning and active learning were alternately implemented until we could not find any relevant records.

### 2.5. Inclusion, Perfomance and Rationality

Because all the algorithms were implemented based on the titles and abstracts, we evaluated the full-texts of the records derived from BiLSTM-based active learning for final inclusion of the articles that met the criteria in Section 2.1. In fact, bibliographic databases might misclassify some records as English journal articles and store their English titles and abstracts.

To verify the performance of the algorithms of text scoring and BiLSTM-active learning, we randomly selected 10% of unlabelled records derived from BiLSTM-based active learning and manually interpreted them as either relevant or irrelevant. This step was iterated three times. Moreover, to verify the rationality of text scoring and BiLSTM-based active learning, we computed the number of relevant records per score rank interval. Generally, the more relevant a record is to the topic in question, the greater the possibility it will receive a high score.

### 2.6. Information Extraction and Analysis

The satellite EO data and landscape factors were extracted manually and synthesized narratively in two ways: (1) charting the dengue landscape factors and their typologies in order to appraise the current situation, regardless of the differences in study areas, methods and materials; (2) tabulating the key characteristics of satellite EO data.

## 3. Results and Discussions

### 3.1. Semi-Supervised Text Classification

Table 2 presents the number of records for each step of semi-supervised text classification. A total of 13,893 bibliographic records were obtained after the broad search, and 7696 records were included after the removal of duplicates. Then, based on text scoring, we identified 2034 possible records, and 131 records were included after the BiLSTM-based active learning that met the inclusion criteria in Section 2.1. Finally, by reading the full texts, we included 101 articles (see more details in Appendix C). The non-English articles (e.g., Chinese, Spanish and Portuguese) and non-journal articles (e.g., book chapters, reviews or conference papers) were excluded.

Table 3 presents the results of each cycle of BiLSTM-based active learning. Evidently, all the relevant records were identified after the fourth cycle. Throughout the process of semi-supervised text classification, we manually evaluated 1056 titles/abstracts (Table 3).

**Figure 2:**
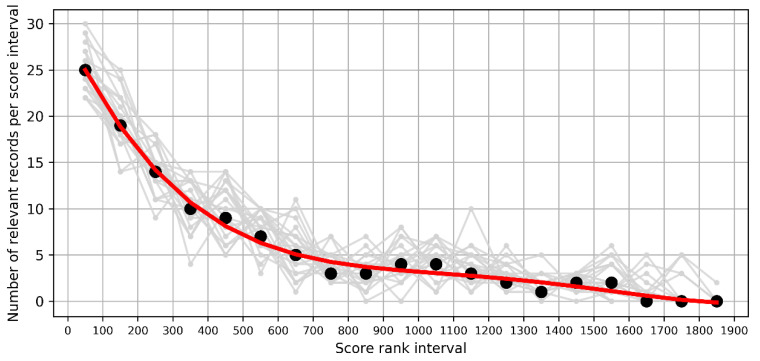
The distribution of the number of relevant records per score rank interval. The grey line represents the distribution of 131 relevant records according to the rank intervals for each of the 20 text scoring experiments, and the red line represents the mean number of relevant records in each score interval.

Moreover, the accurate and rational identification of relevant records can be indicated by the following two facts. First, no relevant records were found by manually evaluating the records selected randomly from the unlabelled dataset (i.e., 925 records after BiLSTM-based active learning). This indicated a good performance of the semi-supervised text classification. Second, although each record probably received different scores in 20 text scoring experiments, the number of relevant records per score rank interval showed a consistent decreasing trend (Figure 2). This indicated the rationality of text scoring using the preset terms and priority levels, that is, the more relevant a record is to the topic question, the greater the possibility it will receive a high score.

The accurate and rational identification of relevant records can be explained by the facts: (1) A clear topic was defined. In fact, modelling or correlating dengue epidemiological or entomological variables with landscape factors in different geographic contexts often includes the identification of landscape factors, landscape characterization and spatio-temporal analysis of dengue cases or vectors. This interdisciplinary topic provides evident features that meet the definition of appropriate inclusion criteria. These criteria then help to define terms and priority levels for text scoring and active learning. (2) The union of the results of 20 text scoring experiments enable the inclusion of potential records as much as possible, and greatly exclude the irrelevant records. (3) BiLSTM has proved to be especially useful in understanding the context of words [23], and active learning based on clear and appropriate inclusion criteria allows for the accurate selection of relevant records and for the control of the balance of positive and negative samples in training datasets for each cycle in BiLSTM learning. Moreover, it should be noted that other models are possible, such as BiLSTM with attention mechanism (AC-BiLSTM) [16] or a combination of CNN and LSTM (C-LSTM) [25], which might generate a high accuracy of text classification.

### 3.2. Dengue Landscape Factors

**Figure 3:**
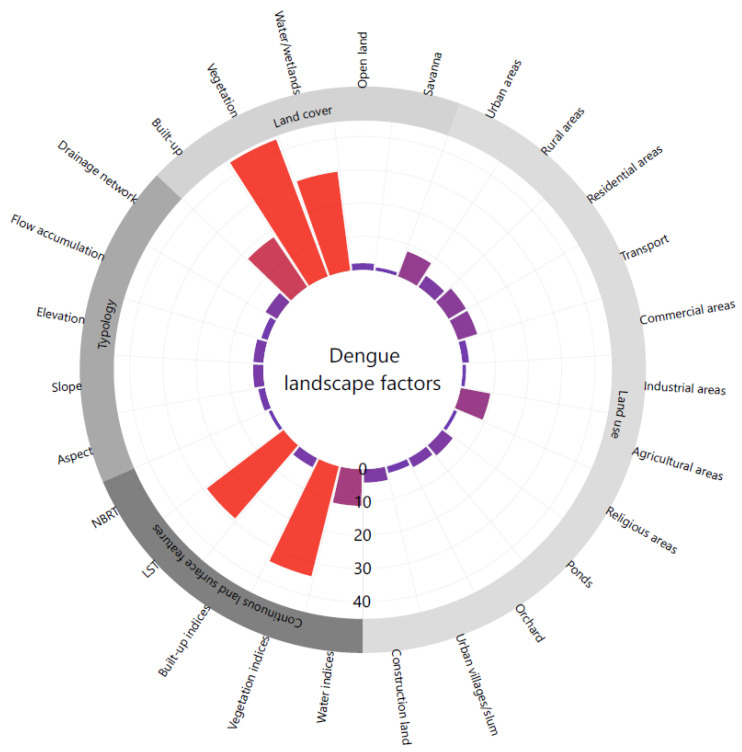
Overview of essential dengue landscape factors derived from the selected articles.

Due to the different study objectives, study areas and spatio-temporal scales, it is difficult to compare the 101 selected studies to find any underlying common viewpoints on the role of landscape factors in dengue transmission. The detailed landscape factors for each study are listed in Table A2. Here, we simply grouped these landscape factors into four categories according to the study [26] (Figure 3):

1. Land cover (LC) refers to the physical and biological cover over the land surface, including built-up areas, vegetation, water/wetlands, open land and savannah. Among them, vegetation often has an association with the vectors’ behaviours and biological cycles, which could be linked with the spatial and temporal dynamics of vectors or the potential resting and breeding sites. Water and wetlands often provide information of places of stagnant water, which are potential breeding sites for dengue vectors.
2. Land use (LU) refers to a territory characterized by current and future planned functional or socio-economic purposes, including agricultural areas, commercial areas, construction areas, industrial areas, ponds, religious areas, residential areas, transport, unused areas, urban areas and rural areas. LU types not only indicate whether the areas are favourable to vector breeding, but also provide information of human behaviour and activities in the areas, the levels of human–*Aedes* encounters, dispersal of mosquitoes and people movement, which are significantly related to dengue epidemics.
3. Topographic factors may provide a proxy of habitat suitability or climate conditions, including elevation, aspect, slope, drainage network, and flow accumulation.
4. Spatially continuous land surface features include spectral indices of vegetation, water and built-up areas (e.g., normalized difference vegetation index (NDVI), enhanced vegetation index (EVI), vegetation fraction index (VFC), normalized difference water index (NDWI), and normalized difference built-up index (NDBI)). Moreover, land surface temperature (LST) refers to a measure of radiative skin temperature of the land surface, which is a significant factor affecting the dengue transmission.

### 3.3. Satellite Earth Observation Data

Among the 101 included articles, only 64 studies used satellite EO data. Table 4 presents the satellite EO sensors, derived products and spatio-temporal resolutions used for identifying dengue landscape factors in selected studies. Evidently, for LU/LC mapping, most studies used very fine (i.e., pixel size < 10 m) and fine (i.e., 10 m ≤ pixel size < 100 m) spatial resolution data, including multi-spectral bands derived from Landsat 4 Thematic Mapper (TM), Landsat 5 TM, Landsat 7 Enhanced Thematic Mapper (ETM+), Landsat 8 Operational Land Imager (OLI), Indian Remote-Sensing Satellite-P6 (IRS-P6), Satellite Pour l’Observation de la Terre 4 (SPOT-4), Sentinel-2, GaoFen-1, SPOT-5, Advanced Land Observing Satellite (ALOS), IKONOS and Quickbird. For topographic factors, two global scale and freely available digital elevation models (DEMs) at resolutions of 30 m and 90 m from the Shuttle Radar Topography Mission (SRTM) and the Advanced Spaceborne Thermal Emission and Reflection Radiometer (ASTER) mission were used to extract topographic features. For continuous land surface features, moderate resolution imaging spectroradiometer (MODIS) products with coarse resolution (i.e., 1000 m ≤ pixel size < 10,000 m) and moderate resolution (i.e., 100 m ≤ pixel size < 1000 m) were widely used to characterize them. In addition, some EO data with fine resolution (i.e., 10 m ≤ pixel size < 100 m) have also made a contribution, such as data from Landsat 5, 7 and 8, SPOT 5 and GeoFen-1.

Although satellite EO sensors and products are pointed out, we do not explain what should be considered while choosing satellite EO data, and making effective use of them. This is an important issue, especially for non-specialized users. Hamm et al. [26] proposed that spatio-temporal scales, uncertainty, spatial quality of EO data and the interaction between uncertainty in EO and disease data should be considered when using EO data for the study of neglected tropical diseases (NTD) (e.g., echinococcosis, schistosomiasis and leptospirosis). This is useful for evaluating EO data in dengue research.

## 4. Possible Future Directions: Landscape Patterns, Satellite Sensors and Deep Learning

### 4.1. In Terms of Landscape Patterns

More in-depth landscape features (e.g., compositional and configurational patterns) could be explored in future studies. Our previous studies characterized forest/non-forest landscapes by computing various landscape metrics and established their links with malaria cases for understanding the contribution of Amazon deforestation on human–vector contact [28,29]. We found very few examples that used landscape metrics in dengue epidemiology, although these metrics have been widely applied in the assessment of LULC changes.

### 4.2. In Terms of Satellite Sensors

LU/LC mapping has continued to be an important research area in recent years, in particular urban LU/LC mapping. Gong et al. [30] proposed the two-level essential urban land use categories (EULUC) and archived the preliminary results of 30 m in China for 2018 using Sentinel-2 images, Luojia night time light data, mobile phone locating request data and point of interests (POI) data. According to our findings (Figure 3), EULUC classes were mostly related to dengue transmission (e.g., residential, commercial, industrial and transportation). Global essential urban land use maps with fine spatial resolution could be useful for landscape-related studies of dengue. Moreover, developing LU/LC maps and integrating them for dengue research in tropical and subtropical regions is difficult due to the presence of clouds and cloud shadows. Synthetic aperture radar (SAR) images could penetrate such barriers and have recently been used for vector-borne disease application [31,32]. However, we found no specific study that used SAR data in dengue research. Third, deep learning frameworks have been increasingly used to predict dengue outbreaks. Many studies have used weather data (e.g., temperature, wind speed, precipitation, humidity), population data and previous dengue cases in deep learning models [33,34].

### 4.3. In Terms of Deep Learning

More recently, one study extracted landscape features (e.g., building, roads, trees, crops, waterway and standing water) from high resolution satellite EO data using CNN models and transfer learning, and added them into time series prediction of dengue outbreaks based on weather data and population density for improving the performance of prediction [35]. This would be a new direction that is practical for identifying the landscape factors with limited labelled data, understanding the landscape–dengue relationships or improving the deep learning-based temporal prediction of dengue risk.

## 5. Conclusions

Satellite EO has been increasingly used in dengue research over the past years, especially for the identification of dengue landscape factors. During that time, various types of landscape factors were considered while the study areas and research objectives have become more complex, and the variety and volume of satellite EO data have been growing over these years. There is an increasing need to know what dengue landscape factors have been studied and what dengue landscape factors have been derived from satellite EO data during the past years. In this study, by integrating the review process, AL mechanism, text scoring and BiLSTM model, we propose a semi-supervised text classification framework that enables the efficient evaluation of bibliographic records derived from bibliographic databases and accurately selects the articles relevant to the research objective. In this study, 101 relevant articles were efficiently selected from bibliographic databases using the proposed approach. Among them, 64 articles used satellite EO data. Valuable information on dengue landscape factors and current satellite EO data was reported. A catalogue of essential dengue landscape factors were identified that were divided into four categories: LU, LC, topography and continuous land surface features. These factors were considered as the direct or indirect proxies of *Aedes* breeding and resting sites, human–Aedes encounters, human mobility and virus replication in dengue transmission. Moreover, future research directions on how to integrate satellite EO data in dengue research were proposed in terms of landscape patterns, satellite sensors and deep learning. This study is an important step towards an efficient method for research evidence synthesis that could be easily applied to other topics, particularly in an interdisciplinary context.

## Figures and Tables

**Table 1:** Pre-set terms and priority levels for titles and abstract scoring.

Priority Levels	Pre-Set Terms (KEYi) Included for Text Scoring	Interval of Weights
High	dengue, environment, landscape, land cover, land use, vegetation, tree, water, built, road, residential, commercial, industrial, normalized difference vegetation index (NDVI), normalized difference water index (NDWI), elevation	[7,10]
Medium	remote sensing, satellite, earth observation	[4,7]
Low	temperature, precipitation	[1,4]

**Table 2:** Number of records derived from each step of semi-supervised text classification.

No.	Semi-Supervised Text Classification Processes	Number of Records
1	Board searches	13,893
2	Removal of duplicates	7696
3	Text scoring	2034
4	Bidirectional long short-term memory (BiLSTM) active learning	131
5	Inclusion	101

**Table 3:** Relevant and unlabeled records derived from BiLSTM-based active learning.

Cycles	BiLSTM	Active Learning		Rest Records
		Relevant	Unlabeled	
Before	--	--	--	2034
1st	599	88	511	1435
2nd	323	39	284	1112
3rd	72	3	69	1036
4th	42	1	41	994
5th	20	0	20	974
Total	1056	131	925	0

**Table 4:** Satellite Earth observation sensors and derived products used for identifying dengue landscape factors. Information on spatial and temporal resolution was taken from Huete et al. [27], Hamm et al. [26] and Marti et al. [9].

Sensors/Products		Variables	Spatial Resolution	Temporal Resolution	Launched/End of Mission
MODIS	MOD11C3	LST	5.5 km	Monthly	2000-02-01 to Present
	MOD13C2	NDVI, VFC	5.5 km	Monthly	2000-02-01 to Present
	MYD11C3	nLST, dLST	5.5 km	Monthly	2002-07-01 to Present
	MYD11A1	LST	1 km	Daily	2002-07-04 to Present
	MOD11A2	LST, nLST, dLST	1 km	8 days	2000-02-18 to Present
	MOD13A3	NDVI, VFC	1 km	Monthly	2000-02-01 to Present
	MOD13C1	NDVI, EVI	500 m	16 days	2000-02-18 to Present
	MCD12Q1	LC	500 m	Yearly	2001-01-01 to 2018-12-31
	MxD09A1	NDVI	250 m	8 days	
	MOD09Q1	NDWI	250 m	8 days	2000-02-24 to Present
	MOD13Q1	NDVI, EVI, LC	250 m	16 days	2000-02-18 to Present
	MYD09GQ	EVI	250 m	Daily	2002-07-04 to Present
AVHRR/2		LST	1.1 km	Daily	1981-06 to 1986-06
SRTM SIR-C	SRTM DEM	Elevation, aspect, slope, drainage, flow accumulation and steam feature	30 m/90 m	-	Released in 2000
ASTER	GDEM	Elevation, drainage	30 m	-	Released in 2009 (v1)
					Released in 2011 (v2)
					Released in 2019 (v3)
Landsat 4 TM		LU/LC	30 m	16 days	1982-07 to 1993-12
Landsat 5 TM		LU/LC, TCB, TCW, TCG, LST, NDVI	30 m	16 days	1984-03 to 2013-06
Landsat 7 ETM+		LU/LC, NDVI, LST, B, G, R, NIR, SWIR1, SWIR2, thermal band	30 m	16 days	1999-04 to Present
Landsat 8 OLI		LU/LC, NDVI, NDWI, NDBI, LST	30 m	16 days	2013-02 to Present
IRS-P6		LC	24 m	5 days	2003-10 to 2013-09
SPOT 4		LU/LC	20 m	2–3 days	1998-03 to 2013-06
Sentinel-2		LC	10 m	10 days	2015-06 to Present (2A)
					2017-03 to Present (2B)
GaoFen-1		LC, NDWI	16 m	≤ 4 days	2013-04 to Present
SPOT 5		LU/LC, NDVI, NDWI	2.5 m, 5 m/10 m	2–3 days	2002-05 to 2015-03
ALOS AVNIR-2		LU/LC	10 m	14 days	1996-08 to 2011-05
ZY-3		LU/LC	2.1 m/5.8 m	5 days	2012-01 to Present
IKONOS		LU	4 m	Approximately 3 days	1999-09 to 2015-03
Quickbird		LU/LC	2.4 m/0.6 m	1–3.5 days	2001-10 to 2015-01
Worldview-2		LC	0.5 m/1.8 m	1.1 days	2009-10 to Present

MODIS: Moderate Resolution Imaging Spectroradiometer; LST: Land Surface Temperature; NDVI: Normalized Difference Vegetation Index; NDBI: Normalized Difference Built-up Index; NDWI: Normalized Difference Water Index; VFC: Vegetation Fractional Coverage; EVI: Enhanced Vegetation Index; AVHRR: Advanced Very High Resolution Radiometer; SRTM: Shuttle Radar Topography Mission; SIR-C: Spaceborne Imaging Radar-C; DEM: Digital Elevation Model; ASTER: Advanced Spaceborne Thermal Emission and Reflection Radiometer; GDEM: Global Digital Elevation Model; TM: Thematic Mapper; ETM+: Enhanced Thematic Mapper; OLI: Operational Land Imager; LU: Land Use; LC: Land Cover; TCB: Tasseled Cap Brightness; TCW: Tasseled Cap Wetness; TCG: Tasseled Cap Greenness; B: Blue band; G: Green band; R: Red band; NIR: Infrared Band; SWIR: Short-wave infrared band; ZY-3: Ziyuan 3; IRS-P6: Indian Remote-Sensing Satellite-P6; SPOT: Satellite Pour l’Observation de la Terre; ALOS: Advanced Land Observing Satellite; AVNIR-2: Advanced Visible and Near Infrared Radiometer type 2.

## Appendix A. Board Searches

**Table A1:** Search terms and number of records derived from bibliographic databases.

No.	Search Terms	WS	SD	Scopus	PubMed
1	dengue AND dwelling	266	7	66	19
2	dengue AND earth observation	13	0	4	3
3	dengue AND habitation	37	3	22	11
4	dengue AND household	618	54	101	282
5	dengue AND land cover	114	4	31	20
6	dengue AND land use	1164	15	120	35
7	dengue AND landscape	179	11	101	70
8	dengue AND precipitation	238	30	175	125
9	dengue AND remote sensing	117	10	56	25
10	dengue AND satellite.	112	11	56	41
11	dengue AND temperature	1976	145	1120	748
12	*Aedes* AND dwelling	733	8	139	52
13	*Aedes* AND earth observation	11	1	3	2
14	*Aedes* AND habitation	88	3	38	14
15	*Aedes* AND household	551	31	430	187
16	*Aedes* AND land cover	266	4	46	39
17	*Aedes* AND land use	3232	19	203	46
18	*Aedes* AND landscape	295	9	153	95
19	*Aedes* AND precipitation	332	21	197	127
20	*Aedes* AND remote sensing	133	0	54	24
21	*Aedes* AND satellite	124	13	68	46
22	*Aedes* AND temperature	3443	171	1616	824

## Appendix B. Word Embedding

Word2Vec [24] is based on deep learning, which could learn grammar and semantic information from a large amount of unlabelled data. Word2Vec Continuous Bag-Of-Words Model (CBOW) model maps each word to a V-dimensional word vector by training, and can calculate the similarity between word vectors to represent the semantic similarity of the text. Word2Vec CBOW architecture predicts the current word based on the context. The input layer here is composed of one-hot encoded input contexts *X1*,...,*Xc*, where the window size is *C*, the glossary size is *V* and the hidden layer is an N-dimensional vector. The final output layer is the output word y that is also encoded by one-hot. The input vector encoded by one-hot is connected to the hidden layer by a *V × N*-dimensional weight matrix *W* and the hidden layer is connected to the output layer by an *N × V* weight matrix *W′*.

## Appendix C. Bidirectional Long Short-Term Memory Model

Generally, LSTM-based RNNs consist of three gates: one input gate it with corresponding weight matrix *W_xi_*, *W_hi_*, *W_ci_*, *bi*; one forget gate *f_t_* with corresponding weight matrix *W_xf_*, *W_hf_*, *W_cf_*_,_ *bf*; one output gate *ot* with corresponding weight matrix *W_xo_*, *W_ho_*, *W_co_*, *bo*. The operation can be summarized as the process of forgetting old information and memorizing new information in the state of the cell, so that information useful for subsequent process operations is passed, and useless information is discarded. The hidden layer state hi is output at each time step. In the process, all gates are set to generate some parameters, using current input *xi*, the state *hi*-1 that the previous step generated, and current state of this cell *ci*-1 (peephole), for the decisions whether to take the inputs, forget the memory stored before, and output the state generated later. The computation can be explained by the following equations:

(A1) $$i_{t}=\sigma \left(W_{xi}x_{t}+W_{hi}h_{t-1}+W_{ci}c_{t-1}+b_{i}\right)$$

(A2) $$f_{t}=\sigma \left(W_{xf}x_{t}+W_{hf}h_{t-1}+W_{cf}c_{t-1}+b_{f}\right)$$

(A3) $$g_{t}=tanh\left(W_{xc}x_{t}+W_{hc}h_{t-1}+W_{cc}c_{t-1}+b_{c}\right)$$

(A4) $$c_{t}=i_{t}g_{t}+f_{t}c_{t-1}$$

(A5) $$o_{t}=\sigma \left(W_{xo}x_{t}+W_{ho}h_{t-1}+W_{co}c_{t}+b_{o}\right)$$

(A6) $$h_{t}=o_{t}tanh\left(c_{t}\right)$$

The BiLSTM uses two independent LSTMs to process the data in both directions and then connects the two final output vectors from both directions.

## Appendix D. List of Articles Derived from the Semi-Supervised Text Classification Framework. Reference List was Alphabetized by the Last Name of the First Author of Each Work. References by the Same Author were Listed Chronologically with the Earliest Work First

**Table A2:** Satellite EO data and dengue landscape factors extracted each of the 101 relevant articles derived from the semi-supervised framework of literature.

ID [Ref.]	First Author/Year	Title	EO Data		Landscape Factors
1 [36]	Acharya et al., 2018	Temporal Variations and Associated Remotely Sensed Environmental Variables of Dengue Fever in Chitwan District, Nepal	MODIS	MOD13C25	NDVI, EVI
			MODIS	MYD11C3	nLST, dLST
9 [37]	Acharya et al., 2018	Modeling the spatially varying risk factors of dengue fever in Jhapa district, Nepal, using the semi-parametric geographically weighted regression model	Landsat 8 OLI/TIRS	Thermal band	LST
			Landsat 8 OLI/TIRS	G, R, NIR, SWIR	NDVI, NDWI, NDBI
2 [38]	Akter et al., 2017	Socio-demographic, ecological factors and dengue infection trends in Australia	-		-
3 [39]	Albrieu-Llinas et al., 2018	Urban environmental clustering to assess the spatial dynamics of Aedes aegypti breeding sites	SPOT 5	Spectral bands	Bare soil, Water, Wetlands, Grass, Tree, Built-up
			Landsat	NIR, SWIR, TIR	NBRT
4 [40]	Ali and Ahmad, 2018	Using analytic hierarchy process with GIS for Dengue risk mapping in Kolkata Municipal Corporation, West Bengal, India	SRTM (SIR-C)	SRTM DEM	Elevation
			Sentinel 2	Spectral bands	Bare soil, Water, Vegetation, Built-up
			Landsat 7 ETM+	Thermal band	LST
5 [41]	Anno et al., 2015	Space-time clustering characteristics of dengue based on ecological, socio-economic and demographic factors in northern Sri Lanka	ALOS/AVNIR-2	B, G, R, NIR	Urbanization ratio
6 [42]	Araujo et al., 2014	Sao Paulo urban heat islands have a higher incidence of dengue than other urban areas	Landsat 5 TM	Thermal band	LST
			Landsat 5 TM	NIR	Vegetation
7 [43]	Arboleda et al., 2009	Mapping Environmental Dimensions of Dengue Fever Transmission Risk in the Aburra Valley, Colombia	SRTM (SIR-C)	SRTM DEM	Elevation, Aspect, Slope
			Landsat 7 ETM +	R, NIR	NDVI
			Landsat 7 ETM +	Spectral bands	B, G, R, NIR, SWIR1, SWIR 2, Thermal band
8 [44]	Arboleda et al., 2011	Spatial and temporal dynamics of Aedes aegypti larval sites in Bello, Colombia	SRTM (SIR-C)	SRTM DEM	Slope, Aspect, Slope
			Landsat 7 ETM +	R, NIR	NDVI
			Landsat 7 ETM +	Spectral bands	B, G, R, NIR, SWIR1, SWIR 2, Thermal band
10 [45]	Ashby et al., 2017	Niche Modeling of Dengue Fever Using Remotely Sensed Environmental Factors and Boosted Regression Trees	MODIS	MYD11A1	nLST, dLST
			MODIS	MYD09GQ	EVI
			SRTM (SIR-C)	SRTM DEM	Elevation
			MODIS	MCD12Q1	Bare soil, Cropland, Forest, Savanna, Urban, Wetlands, Shrubland
11 [46]	Attaway et al., 2016	Risk analysis for dengue suitability in Africa using the ArcGIS predictive analysis tools (PA tools)	-	-	-
12 [47]	Aziz, S. et al. (2014)	Spatial density of Aedes distribution in urban areas: A case study of Breteau index in Kuala Lumpur, Malaysia	SPOT 5	-	Water, Built-up, Sparse vegetation, Dense vegetation, Cleared area
13 [48]	Beilhe, Leila Bagny et al. (2012)	Spread of invasive Aedes albopictus and decline of resident Aedes aegypti in urban areas of Mayotte 2007–2010	-	-	-
14 [49]	Bett, Bernard et al. (2019)	Spatiotemporal analysis of historical records (2001–2012) on dengue fever in Vietnam and development of a statistical model for forecasting risk.	MODIS	MCD12Q1	Forest, Woodland, Grass, Shrub, Cropland, Built-up, Wetlands
15 [50]	Bhardwaj et al. (2012)	Developing a statistical dengue risk prediction model for the state of Delhi based on various environmental variables	Landsat 7 ETM+	-	Built-up, Vegetation
16 [51]	Buczak et al. (2012)	A data-driven epidemiological prediction method for dengue outbreaks using local and remote sensing data	MODIS	-	NDVI, EVI
17 [52]	Buczak et al. (2014)	Prediction of High Incidence of Dengue in the Philippines	MODIS	-	NDVI, EVI
18 [53]	Butt et al. (2019)	Towards a Web GIS-based approach for mapping a dengue outbreak	Landsat 5 TM	TIR	LST
			Landsat 5 TM	R, NIR	NDVI
			Landsat 5 TM	Spectral bands	Built-up, Vegetation, Water, Bare soil, Mixed areas
19 [54]	Cao et al. (2017)	Individual and Interactive Effects of Socio-Ecological Factors on Dengue Fever at Fine Spatial Scale: A Geographical Detector-Based Analysis	MODIS	MOD13A3	NDVI, VFC
			Landsat 8 OLI/Quickbird	-	Urban villages
20 [55]	Carbajo et al. (2001)	Dengue transmission risk maps of Argentina	-	-	-
22 [56]	Chen et al. (2018)	Neighborhood level real-time forecasting of dengue cases in tropical urban Singapore	-	-	-
21 [57]	Chen et al. (2019)	Spatiotemporal Transmission Patterns and Determinants of Dengue Fever: A Case Study of Guangzhou, China	SPOT 5/Baidu map	Panchromatic and spectral bands	Road, Subway, Ponds, Residential areas
23 [58]	Cheong et al. (2014)	Assessment of land use factors associated with dengue cases in Malaysia using Boosted Regression Trees	Landsat 7 ETM +/SPOT 4	-	Residential areas, Agricultural areas, Forest, Water, Mixed horticulture, Open land, Rubber, Oil palm, Swamp forest, Mining, Orchard
24 [59]	Chiu et al. (2014)	A probabilistic spatial dengue fever risk assessment by a threshold-based-quantile regression method	-	-	-
25 [60]	Chuang et al. (2018)	Epidemiological Characteristics and Space-Time Analysis of the 2015 Dengue Outbreak in the Metropolitan Region of Tainan City, Taiwan	-	-	-
26 [61]	Cox et al. (2007)	Habitat segregation of dengue vectors along an urban environmental gradient	Landsat 7 ETM +	-	Urban, Suburban, Rural, Forest, High density housing, Low density housing
27 [62]	Dhewantara, Pandji Wibawa et al. (2019)	Spatial and temporal variation of dengue incidence in the island of Bali, Indonesia: An ecological study	ASTER	GDEM	Elevation
28 [63]	Dom et al. (2013)	Coupling of remote sensing data and environmental-related parameters for dengue transmission risk assessment in Subang Jaya, Malaysia	IKONOS	-	Residential areas, Industrial areas, Commercial areas, Open area
30 [64]	Espinosa et al.(2016)	Temporal Dynamics and Spatial Patterns of Aedes aegypti Breeding Sites, in the Context of a Dengue Control Program in Tartagal (Salta Province, Argentina)	SPOT 5	Spectral bands	Water, High vegetation, Low vegetation, Cropland, Bare soil, Urban area
29 [65]	Espinosa et al., 2018	Operational satellite-based temporal modelling of Aedes population in Argentina	MODIS	MOD13Q1	NDVI, NDWI
			MODIS	MOD11A2	dLST, nLST
31 [66]	Estallo et al. (2016)	MODIS Environmental Data to Assess Chikungunya, Dengue, and Zika Diseases Through Aedes (Stegomia) aegypti Oviposition Activity Estimation	MODIS	MOD13Q1	NDVI
			MODIS	MOD11A2	dLST
32 [67]	Fareed et al. (2016)	Spatio-Temporal Extension and Spatial Analyses of Dengue from Rawalpindi, Islamabad and Swat during 2010–2014	ASTER	GDEM	Elevation, Drainage network
			Landsat 4 TM, Landsat 5 TM, Landsat 7 ETM+, and Landsat 8 OLI	Spectral bands	Bare soil, Built-up, Water, Vegetation, Construction area
33 [68]	Fatima, Syeda Hira et al. (2016)	Species Distribution Modelling of Aedes aegypti in two dengue-endemic regions of Pakistan	SRTM (SIR-C)	SRTM DEM	Elevation
			Landsat 8 OLI	-	Vegetation, Water, Built-up, Road
35 [69]	Fuller et al. (2009)	El Nino Southern Oscillation and vegetation dynamics as predictors of dengue fever cases in Costa Rica.	MODIS	MOD13C1	EVI, NDVI
34 [70]	Fuller et al. (2010)	Dengue vector (Aedes aegypti) larval habitats in an urban environment of Costa Rica analysed with ASTER and QuickBird imagery	Quickbird	-	Built-up, Tree
36 [71]	Garcia et al. (2011)	An examination of the spatial factors of dengue cases in Quezon City, Philippines: A Geographic Information System (GLS)-based approach, 2005–2008	-	-	-
37 [72]	German et al. (2018)	Exploring satellite based temporal forecast modelling of Aedes aegypti oviposition from an operational perspective	MODIS	MOD13Q1	NDVI, NDWI,
			MODIS	MOD11A2	nLST, dLST
38 [73]	Hira et al. (2018)	Patterns of occurrence of dengue and chikungunya, and spatial distribution of mosquito vector Aedes albopictus in Swabi district, Pakistan	SRTM (SIR-C)	SRTM DEM	Elevation
39 [74]	Huang et al. (2018)	Spatial Clustering of Dengue Fever Incidence and Its Association with Surrounding Greenness	MODIS	MxD09A1	NDVI
40 [75]	Husnina et al. (2019)	Forest cover and climate as potential drivers for dengue fever in Sumatra and Kalimantan 2006–2016: a spatiotemporal analysis	-	-	-
41 [76]	Kesetyaningsi et al. (2018)	Determination of environmental factors affecting dengue incidence in Sleman District, Yogyakarta, Indonesia	Quickbird	-	Vegetation
43 [77]	Khalid and Ghaffar. (2014)	Dengue transmission based on urban environmental gradients in different cities of Pakistan	SRTM (SIR-C)	SRTM DEM	Flow accumulation, Stream feature, Drainage density
			SPOT 5/Landsat TM	Spectral bands	Urban area, Bare soil, Forest, Water, Vegetation, Wedged land, Waterlogged land, Dry bare land, Rocky bare land, Deserted land
42 [78]	Khalid and Ghaffar. (2015)	Environmental risk factors and hotspot analysis of dengue distribution in Pakistan	SRTM (SIR-C)	SRTM DEM	Drainage
44 [79]	Khormi and Kumar. (2011)	Modeling dengue fever risk based on socioeconomic parameters, nationality and age groups: GIS and remote sensing based case study	SPOT 5	-	Quality of neighborhood
45 [80]	Koyadun et al. (2012)	Ecologic and sociodemographic risk determinants for dengue transmission in urban areas in Thailand.	-	-	-
46 [81]	Lana et al. (2017)	The introduction of dengue follows transportation infrastructure changes in the state of Acre, Brazil: A network-based analysis	-	-	-
47 [82]	Landau and Leeuwen. (2012)	Fine scale spatial urban land cover factors associated with adult mosquito abundance and risk in Tucson, Arizona	NAIP aerial image/LiDAR elevation	Spectral bands	Bare soil, Pavement, Structure, Pool, Water (ponds and lakes), Grass, Shrub, Tree
48 [83]	Lee et al. (2019)	Human Activities Attract Harmful Mosquitoes in a Tropical Urban Landscape.	-	-	-
49 [84]	Li et al. (2013)	Abiotic Determinants to the Spatial Dynamics of Dengue Fever in Guangzhou	MODIS	MOD13Q1	Cropland, Built-up, Construction area, Vegetation, Water
50 [85]	Lian, Cheah Whye et al. (2006)	Spatial, environmental and entomological risk factors analysis on a rural dengue outbreak in Lundu District in Sarawak, Malaysia	-	-	-
51 [86]	Lippi et al. (2019)	Geographic shifts in Aedes aegypti habitat suitability in Ecuador using larval surveillance data and ecological niche modeling: Implications of climate change for public health vector control	-	-	-
52 [87]	Little et al. (2011)	Co-occurrence Patterns of the Dengue Vector Aedes aegypti and Aedes mediovitattus, a Dengue Competent Mosquito in Puerto Rico	WorldView 2	Spectral bands	Bare soil, Grass, Scrub, Tree, Urban area
53 [88]	Little et al. (2017)	Local environmental and meteorological conditions influencing the invasive mosquito Ae. albopictus and arbovirus transmission risk in New York City	-	-	-
54 [89]	Little et al. (2017)	Socio-Ecological Mechanisms Supporting High Densities of Aedes albopictus (Diptera: Culicidae) in Baltimore, MD	Landsat	R, NIR	NDVI
55 [90]	Liu et al. (2018)	Spatiotemporal patterns and determinants of dengue at county level in China from 2005–2017	-	-	-
56 [91]	Lozano-Fuentes et al. (2012)	The Dengue Virus Mosquito Vector Aedes aegypti at High Elevation in Mexico	-	-	-
57 [5]	Machault et al., 2014	Mapping Entomological Dengue Risk Levels in Martinique Using High-Resolution Remote-Sensing Environmental Data	Geoeye-1	Spectral bands	NDVI, MNDWI, ANDWI
			Geoeye-1	Spectral bands	Sparsely vegetated soil, Grass, Asphalt
58 [92]	Mahabir et al. (2012)	Impact of road networks on the distribution of dengue fever cases in Trinidad, West Indies	-	-	-
59 [93]	Mahmood et al. (2019)	Spatiotemporal analysis of dengue outbreaks in Samanabad town, Lahore metropolitan area, using geospatial techniques	-	-	-
60 [94]	Mala and Jat. (2018)	Implications of meteorological and physiographical parameters on dengue fever occurrences in Delhi	Landsat 7 ETM+, Landsat 8 OLI, IRS-P6, Sentinel-2	Panchromatic and spectral bands	Built-up, Water, Vegetation
61 [95]	Martinez-Bello et al. (2017)	Spatiotemporal modeling of relative risk of dengue disease in Colombia	MODIS	MOD11A2	LST
			MODIS	MOD13Q1	NDVI
62 [96]	Martinez-Bello et al. (2017)	Relative risk estimation of dengue disease at small spatial scale	MODIS	MOD11A2	LST
			Landsat 7 ETM+, Landsat 8 OLI	R, NIR	NDVI
63 [97]	McClure et al. (2018)	Land Use and Larval Habitat Increase Aedes albopictus (Diptera: Culicidae) and Culex quinquefasciatus (Diptera: Culicidae) Abundance in Lowland Hawaii	Quickbird	-	Developed land
64 [98]	Messina et al. (2019)	The current and future global distribution and population at risk of dengue	-	-	-
65 [99]	Murdock et al. (2017)	Fine-scale variation in microclimate across an urban landscape shapes variation in mosquito population dynamics and the potential of Aedes albopictus to transmit arboviral disease	-	-	-
66 [100]	Nakhapakorn and Tripathi. (2005)	An information value based analysis of physical and climatic factors affecting dengue fever and dengue haemorrhagic fever incidence	Landsat TM	-	Agricultural areas, Forest, Water, Built-up
67 [101]	Nejati et al. (2017)	Potential Risk Areas of Aedes albopictus in South-Eastern Iran: A Vector of Dengue Fever, Zika, and Chikungunya	ASTER	ASTER DEM	Elevation
			Landsat 8 OLI	R, NIR	NDVI
			Landsat 8 OLI	Spectral bands	Water, Urban area (residential), Rural area (residential)
68 [102]	Nitatpattana et al. (2007)	Potential association of dengue hemorrhagic fever incidence and remote senses land surface temperature, Thailand, 1998	National Oceanic and Atmospheric Administration-14	-	LST
69 [103]	Ogashawara et al. (2019)	Spatial-Temporal Assessment of Environmental Factors Related to Dengue Outbreaks in São Paulo, Brazil	Landsat 8 TIRS	Thermal bands	LST
			Landsat 8 OLI	Spectral bands	NDVI, NDWI, NDBI
70 [104]	Pineda-Cortel et al. (2019)	Modeling and predicting dengue fever cases in key regions of the Philippines using remote sensing data	MODIS	MOD11C3	nLST, dLST
			MODIS	MOD13Q1	NDVI
71 [105]	Qu et al. (2018)	Effects of socio-economic and environmental factors on the spatial heterogeneity of dengue fever investigated at a fine scale	-	-	-
72 [106]	Qureshi et al. (2017)	The distribution of Aedes aegypti (diptera, culicidae) in eight selected parks of Lahore, using oviposition traps during rainy season	-	-	-
73 [107]	Rahm et al. (2016)	Forecasting of Dengue Disease Incident Risks Using Non-stationary Spatial of Geostatistics Model in Bone Regency Indonesia	-	-	-
74 [108]	Ren et al. (2019)	Urban villages as transfer stations for dengue fever epidemic: A case study in Guangzhou, China	ZY-3	Panchromatic and spectral bands	Normal construction areas, Urban villages, Water, Vegetation, Unused land
75 [109]	Restrepo et al. (2014)	National spatial and temporal patterns of notified dengue cases, Colombia 2007–2010	-	-	-
76 [110]	Richards et al. (2006)	Spatial analysis of Aedes albopictus (Diptera: Culicidae) oviposition in suburban neighborhoods of a piedmont community in North Carolina	-	-	-
77 [111]	Rogers et al. (2014)	Using global maps to predict the risk of dengue in Europe	MODIS	-	nLST, dLST
			MODIS	-	NDVI, EVI
78 [112]	Rosa-Freitas et al. (2010)	Dengue and land cover heterogeneity in Rio de Janeiro	-	-	-
79 [113]	Rotela et al. (2007)	Space-time analysis of the dengue spreading dynamics in the 2004 Tartagal outbreak, Northern Argentina	Landsat 5 TM	Spectral bands	Road, River, Street, Vegetation
			Landsat 5 TM	Spectral bands	TCB, TCG, TCW, Landsat bands (7 to 13)
80 [114]	Saravanabavan et al. (2019)	Identification of dengue risk zone: a geo-medical study on Madurai city	-	-	-
82 [115]	Sarfraz et al. (2012)	Analyzing the spatio-temporal relationship between dengue vector larval density and land-use using factor analysis and spatial ring mapping	-	-	-
81 [116]	Sarfraz et al. (2014)	Near real-time characterisation of urban environments: a holistic approach for monitoring dengue fever risk areas	ALOS AVNIR-2	Spectral bands	Built-up, Vegetation, Water, Bare soil, Road, Institution, Religious areas, Market
83 [117]	Sarfraz et al. (2014)	Mapping urban and peri-urban breeding habitats of Aedes mosquitoes using a fuzzy analytical hierarchical process based on climatic and physical parameters	SRTM (SIR-C)	SRTM DEM	Elevation
			MODIS	MYD11C3	dLST, nLST
84 [118]	Scavuzzo et al. (2018)	Modeling Dengue vector population using remotely sensed data and machine learning	MODIS	MOD13Q1	NDVI, NDWI
			MODIS	MOD11A2	nLST, dLST
85 [119]	Shafie (2011)	Evaluation of the Spatial Risk Factors for High Incidence of Dengue Fever and Dengue Hemorrhagic Fever Using GIS Application	-	-	-
86 [120]	Sheela et al. (2015)	Assessment of changes of vector borne diseases with wetland characteristics using multivariate analysis	IRSP6 LISSIII	-	Wetlands, Inland areas, Inland waterlogged areas, Inland river, Inland man made ponds, Inland reservoirs, Coastal lagoons, Coastal beaches and creek, Aquatic vegetation, Turbidity
87 [121]	Sheela et al. (2017)	Assessment of relation of land use characteristics with vector-borne diseases in tropical areas	-	-	-
88 [122]	Stanforth et al., (2016)	Exploratory Analysis of Dengue Fever Niche Variables within the Rio Magdalena Watershed	MODIS	MYD11A1	LST
			MODIS	MYD09GQ	EVI
			SRTM (SIR-C)	SRTM DEM	Elevation
			MODIS	MCD12Q1	Bare soil, Cropland, Forest, Urban
89 [123]	Tariq and Zaidi. (2019)	Geostatistical modeling of dengue disease in Lahore, Pakistan	SPOT 5	Spectral bands	NDVI, NDWI
			Landsat 5 TM	Spectral bands	LST
90 [124]	Teurlai et al. (2015)	Socio-economic and Climate Factors Associated with Dengue Fever Spatial Heterogeneity: A Worked Example in New Caledonia	-	-	-
91 [125]	Tian et al. (2016)	Surface water areas significantly impacted 2014 dengue outbreaks in Guangzhou, China	Landsat 4 TM, Landsat 5 TM, Landsat 7 ETM+, and Landsat 8 OLI	Spectral bands	Water
92 [126]	Tiong et al. (2015)	Evaluation of land cover and prevalence of dengue in Malaysia	-	-	-
93 [127]	Tipayamongkholgul and Lisakulruk. (2011)	Socio-geographical factors in vulnerability to dengue in Thai villages: a spatial regression analysis	-	-	-
94 [128]	Troyo et al. (2009)	Urban structure and dengue fever in Puntarenas, Costa Rica.	MODIS	-	EVI
			ASTER	Spectral bands	NDVI
			Quickbird	Panchrmatic and spectral bands	Built-up, Tree
95 [129]	Tsuda et al. (2006)	Different spatial distribution of Aedes aegypti and Aedes albopictus along an urban-rural gradient and the relating environmental factors examined in three villages in northern Thailand	-	-	-
96 [130]	Van Benthem et al. (2005)	Spatial patterns of and risk factors for seropositivity for dengue infection	Landsat 2000	-	Vegetation, Built-up, Cropland
97 [131]	Vanwambeke et al. (2006)	Multi-level analyses of spatial and temporal determinants for dengue infection	Landsat 2000	-	Orchard, Water, Bare soil, Village areas, Agricultural areas
98 [132]	Vezzani et al. (2005)	Detailed assessment of microhabitat suitability for Aedes aegypti (Diptera: Culicidae) in Buenos Aires, Argentina	-	-	-
99 [133]	Wiese et al. (2019)	Integrating environmental and neighborhood factors in MaxEnt modeling to predict species distributions: A case study of Aedes albopictus in southeastern Pennsylvania	MODIS	MOD13Q1	EVI
			MODIS	MOD09Q1	NDWI
			SRTM (SIR-C)	SRTM DEM	Elevation, Slope, Flow accumulation
100 [134]	Yue et al. (2018)	Spatial analysis of dengue fever and exploration of its environmental and socio-economic risk factors using ordinary least squares: A case study in five districts of Guangzhou City, China, 2014	GaoFen-1	Spectral bands	NDWI
			GaoFen-1	Spectral bands	Water, Vegetation, Built-up
			MODIS	MOD11A2	nLST, dLST
101 [135]	Zheng et al. (2019)	Spatiotemporal characteristics and primary influencing factors of typical dengue fever epidemics in China	-	-	-
